# Treating Fear of Cancer Recurrence with Eye Movement Desensitization Reprocessing: A Sequential, Randomized Single-Case Experimental Design

**DOI:** 10.17505/jpor.2025.27699

**Published:** 2025-04-01

**Authors:** Pascalle A. I. Van der Wolf, Melanie P. J. Schellekens, Marije L. van der Lee

**Affiliations:** a Scientific Research Department, Helen Dowling Institute, Centre for Psycho-Oncology, Bilthoven, The Netherlands; b Department of Medical and Clinical Psychology, Tilburg University School of Social and Behavioral Sciences, Tilburg, The Netherlands

**Keywords:** Fear of cancer recurrence, Eye Movement Desensitization Reprocessing, Single Case Experimental Design, Intrusions, Death anxiety

## Abstract

Fear of cancer recurrence (FCR) is defined as “fear, worry, or concern relating to the possibility that cancer will come back or progress”. After cancer treatment, 20% of patients suffer from clinical fear of cancer recurrence (FCR), warranting specialized treatment. While intrusive catastrophic scenarios are clinical symptoms of FCR, they are rarely the key focus in current FCR treatments. Eye Movement Desensitization Reprocessing (EMDR) including the flash forward procedure explicitly addresses these intrusions. The present study explored whether EMDR is effective in treating clinical FCR. A sequentially replicated, randomized single-case experimental design was used among six cancer survivors with clinical levels of FCR. During an 84-day period, participants daily registered their FCR level. The Fear of Recurrence Inventory was administered at baseline, EMDR start, EMDR completion and study completion. The start of EMDR was randomized. All participants commented positively on the effect of EMDR during the semi-structured interviews: EMDR helped decrease intrusions and face death anxiety. Visual analysis of daily FCR were in line with these comments. Regression analysis showed a significant decrease of daily FCR in two participants, while the randomization test showed no effects. FCRI scores decreased below clinical levels in all participants, which was considered a reliable change in four participants. There was no drop-out. In light of these mixed findings, EMDR appears a promising treatment for FCR. Further research needs to establish its effectiveness and explore whether diminishing the emotional load of intrusions constitutes the working mechanism of EMDR in FCR.

## Introduction

Fear of cancer recurrence (FCR) is defined as “fear, worry, or concern relating to the possibility that cancer will come back or progress” (Lebel et al., 2016). FCR is described by survivors as always present in the back of one’s mind and triggered by internal (e.g., pain in the body) and external events (e.g., waiting for scan results) (Almeida et al., 2019). Low levels of FCR are considered normal and potentially adaptive for survivors because it can facilitate adopting a healthy lifestyle, vigilance towards potential signs of recurrence and adherence to medical follow-up (Fardell et al., 2016). Almost 20% of cancer survivors experience severe or clinical levels of FCR (Luigjes-Huizer et al., 2022). Symptoms of clinical FCR include (1) high levels of

preoccupation, including intrusive thoughts/images, and (2) high levels of worry (3) that are persistent, and (4) hypervigilance to bodily symptoms (Mutsaers et al., 2020), impairing daily life activities and the ability to plan for the future (Almeida et al., 2019). Patients with clinical FCR make increased use of healthcare services and report a lower quality of life (Williams, et al, 2021). Clinical FCR often presents with comorbidities like anxiety disorders, stress-related disorders, and mood disorders (Bisseling et al., 2021). Psychological care is warranted, as FCR does not appear to decrease without psychological treatment (Simard et al., 2013).

A transdiagnostic model of the etiology and maintenance of FCR posits that a cancer diagnosis triggers a realistic life threat and confronts the individual with the limits of human control (Curran et al., 2017). Testing their model (*n*=211), Curran and colleagues (2020) identified four factors that explained FCR: intrusions, death anxiety, threat appraisals and beliefs about worry. Long-term avoidance of death anxiety by suppression or denial often corresponds with intrusive thoughts, or other fears (Sharpe et al., 2018).

An extensive meta-analysis showed that different types of psychological treatment have been effective in reducing FCR (Tauber et al., 2019). However, a substantial proportion of patients remain in the clinical range of FCR after treatment. Moreover, the currently available treatments for FCR are not explicitly aimed at intrusions of illness-related threatening moments from the past (e.g., hearing the diagnosis, experiencing threatening medical complications) nor at intrusions of scenarios in the future causing death anxiety (e.g., final stage of disease, impact of one’s death on loved ones).

Eye Movement Desensitization and Reprocessing (EMDR) has a strong focus on intrusive images. EMDR is an eclectic form of psychological therapy, targeting traumatic memories and associated distress (Shapiro, 1989). EMDR is effective in treating post-traumatic stress disorder (PTSD), panic disorder and obsessive-compulsive disorder (Horst et al., 2017; Yunitri et al., 2020). In cancer survivors EMDR has also been effective in treatment of PTSD and appears promising in treating psychological distress and anxiety (Jarero et al., 2018; Tarquinio et al., 2022). In EMDR, a dual-task approach is used by focusing on the traumatic memory, while simultaneously focusing on external stimulation, such as following a visual cue (Shapiro, 2001). This dual-task approach creates competition in one’s working memory and as a result, disruptive images become less vivid and emotional (Engelhard et al., 2010). Often, a spontaneous process of altered meaning assignment takes place, which helps to process these traumatic memories in one’s personal history (De Jongh & Ten Broeke, 2019

While most EMDR protocols are focused on desensitizing disruptive traumatic memories, the desensitization effect of the dual-task approach is also effective for most feared catastrophic future events, so-called ‘flash forwards’ (FF) (Engelhard et al., 2011; Logie & De Jongh, 2018; Horst et al., 2017). An EMDR protocol including FF for cancer survivors, will target three of the four key predictors of clinical FCR (i.e., intrusions, death anxiety, and threat appraisal), and subsequently reduce the level of FCR (Curran et al., 2020). The aim of the present study was to explore whether an EMDR protocol with FF is effective in treating FCR.

## Methods

### Study Design

A sequentially replicated, randomized single-case A-B-phase design (Heyvaert & Onghena, 2014) was used to explore whether EMDR is helpful in treating FCR. This study was approved by the local medical ethics committee METC Brabant (P2104). The total number of measurements for each participant was 84 daily measurements (12 weeks).

Phase A consisted of a baseline period in which no intervention was offered. Phase B consisted of an intervention period, in which EMDR treatment was offered plus a followup period until the end of the twelve weeks. By comparing daily repeated measurements of FCR between the phases, we evaluated the effect of the intervention. If FCR changed from the baseline to treatment phase, it can be assumed that EMDR was responsible for this change. To control for history and maturation effects (Heyvaert & Onghena, 2014), the start of phase B was randomized in a restricted time frame. Following Kratochwill’s recommendations (2010), we decided on a baseline period of at least 7 days with a maximum of 37 days. This ensured that the start of the intervention phase could be randomized over 30 measurement moments and the intervention phase would be of sufficient duration to complete the EMDR treatment, which was expected to last 3 to 5 weeks. To further strengthen the internal validity of the A-B-phase design, the study aimed to replicate this over seven individuals (Kratochwill & Levin, 2010).

### Participants

The single-case experiments were implemented in the routine clinical care of the Helen Dowling Institute (HDI), a mental healthcare institute for cancer patients and their close others. Patients on the waiting list suffering from FCR were informed about the study and screened for eligibility. Inclusion criteria were having received medical treatment for cancer but no longer in the acute phase of treatment, being 18 years or older, sufficient understanding of Dutch language, and scoring >22 on the severity subscale of the Fear of Cancer Recurrence Inventory (FCRI-SF). Exclusion criteria were acute suicidal threat, acute psychotic disorder, and severe early traumatic experiences. At the start of EMDR, therapists checked again whether participants did not meet any of the exclusion criteria.

### Procedure

After participants provided written informed consent, the day of the first EMDR session was determined randomly by the computer. The start could be adjusted for one or two days due to weekends, or immovable appointments of participants. Participants daily registered their level of FCR between 18.30 and 21.30 via an app (ethicadata.com) on their phone. FCR was also examined with the FCRI-SF at study commencement (t0), start of EMDR (t1), after last EMDR session (t2), and study completion (t3). At the end of the 84 days participants and therapists were interviewed to explore their experiences with EMDR.

### Measures

#### Sociodemographic data

Medical and sociodemographic data were extracted from patients’ files.

#### Daily level of FCR and Enjoyment

FCR was assessed with the question “In the past 24 hours I was afraid that the cancer would come back,” which could be answered on a visual analogue scale (VAS), ranging from not at all (0) to very much afraid (100). To balance this question with a positive item, we also assessed enjoyment with “In the past 24 hours I experienced enjoyment” which was answered on a VAS, ranging from not at all (0) to a lot of enjoyment (100).

#### FCR severity

The 9-item FCRI-SF is part of the FCRI and assesses FCR severity. Items are scored on a 5-point likert scale, ranging from 0 (not at all) to 4 (a great deal). Scores range from 0 to 36, and a score of >22 indicates clinical severity of FCR. The FCRI-SF and its Dutch translation show good psychometric values (Simard & Savard, 2009; van Helmondt et al., 2017).

#### Semi-structured interviews

Semi-structured interviews were conducted by the first author, who has novice experience with interviewing for qualitative research but extensive experience with various psychological interview techniques. Participants were asked about their expectations of EMDR, what changes in FCR and daily functioning they attributed to EMDR, events that might have affected FCR during the treatment period and feedback on EMDR. The interviewer explicitly asked for negative experiences with EMDR when participants did not address these themselves. All interviews were either face-to-face or via secured videocalls and were recorded and transcribed verbatim.

#### EMDR

Following the Dutch EMDR protocol (De Jongh & Ten Broeke, 2019; Hornsveld et al., 2018), a three-step approach of processing was applied: targeting (1) disturbing memories of past experiences causing FCR; (2) catastrophic images fueling death anxiety, or so-called FFs (e.g. images of loved ones in despair after one’s death); and (3) triggers that currently evoke distress. For the first step, memories of etiological and/or aggravating events of FCR were selected, activated and reprocessed by asking the participant to bring up the memory and to concentrate on the most disturbing image of the memory, a self-referencing dysfunctional belief, and its emotional and somatic components. Next, the clinician instructed the participant to concentrate on these elements of the memory while simultaneously making rapid eye movements following a visual stimulus. If necessary, the therapist added working memory taxation by using faster eye movements, earphones with a clicking sound, or hand-holdable pulsers providing alternating tactile stimulation (De Jongh et al., 2013). Participants were asked to report their associative thoughts after each set (± 30 sec) of working memory taxation, which were repeated for 5-10 minutes, after which the client rated the subjective units of distress (SUD). The desensitization series were repeated until the session ended or when the SUD-rating decreased to zero. A similar desensitization procedure was applied to the FF in the second step.

The third step was optional; its suitability was assessed by the therapist. If certain situations or triggers were avoided, a mental videocheck was made in which desensitization was applied. If necessary, a behavioral experiment could be added. All sessions lasted 90 minutes and were offered once a week.

#### EMDR therapists

EMDR treatment was offered by four registered psychologists, who were all trained by the Dutch EMDR society. Three therapists were EMDR practitioners, and one therapist had basic EMDR training. They were well experienced with using EMDR in oncology patients prior to the study. They were trained and supervised by the first author to apply the EMDR protocol for FCR.

### Data Analysis

### Sample size justification

Following the simulation study by Heyvaert et al (2017), it was concluded that 84 daily assessments and 30 randomization starting points of treatment replicated over 7 participants would be sufficient to reach statistical power of at least 80%. Following Single Case Experimental Design (SCED) guidelines, we used several statistical methods specifically recommended for SCED to examine an intervention effect (Michiels & Onghena, 2018; Onghena et al., 2019).

### Visual analysis

We conducted visual analysis, inspecting the observed data for changes in level, trends, variability, immediacy of treatment effect, phase overlap and consistency of data patterns across similar phases (Kratochwill et al., 2010).

### Regression analysis

To test the intervention effect in individual participants, we conducted ordinary least square regression analysis (OLS) (Huitema & McKean, 1998). To explore the average treatment effect in all participants, hierarchical linear modelling (HLM) was used (Manolov & Moeyaert, 2017; van den Noortgate & Onghena, 2003). By treating data as ‘nested in individuals,’ the shared variance in each participant can be accounted for in HLM (van den Noortgate & Onghena, 2003). OLS and HLM were performed using the Multi SCED software tool, which has been specifically designed for SCED data (Declercq et al., 2020).

### Randomization test

The Randomization Test (RT), a non-parametric approach (Bulté & Onghena, 2009; Heyvaert & Onghena, 2014) is a permutation test which involves calculating the test statistic (i.e., mean difference) between the phases for each possible assignment in the randomization period and examining where the observed test statistic falls within the distribution of all possible test statistic values. The proportion of test statistic values that is as extreme, or even more extreme than the observed difference in means is the *p*-value of the RT (Heyvaert & Onghena, 2014). This RT was performed using the Single Case Data Analysis (Shiny SCDA) software, version 2.8. (Bulté & Onghena, 2013). In addition, a combined *p*-value was calculated considering all participants simultaneously in a meta-analysis, using Edgington’s additive method (Manly, 2007).

### Reliable change index (RCI)

We calculated the RCI (Jacobson & Truax, 1991) between the pre-treatment (T1) and follow-up (T3) scores of the FCRI-SF. We used the validation study of the Dutch FCRI - SF to calculate the RCI, in which the standard deviation of the second assessment was 6.7 and the test-retest intraclass correlation coefficient was 0.87 (van Helmondt et al., 2021). If the RCI indicated a reliable change and the (T3) score decreased below the cutoff of clinical FCR (i.e., <22), the decrease in FCR was considered clinically relevant.

### Qualitative data analysis

We used the inductive thematic analysis approach (Braun & Clarke, 2006) to explore patients’ experiences with EMDR. The first author familiarized herself with the transcripts, conducted initial open coding of the interviews and together with the other authors organized the codes into themes.

## Results

### Participants

Between March and September 2021, 35 patients were invited to participate in the study. Three patients did not meet the inclusion criteria and 25 preferred regular treatment because of practical reasons (holidays, distance to HDI) or severe comorbid problems which they preferred to address first (e.g., relational problems, fatigue, depressive symptoms). Seven participants were included, of which one had to be excluded from the analysis because of unexpected interim medical treatment. The remaining six participants were all women, with a mean age of 43.3 years (*SD*=9.5). Participants were diagnosed with Anxiety Not Otherwise Specified (*n*=3), Post-Traumatic Stress Disorder (*n*=2) and Somatic Symptom Disorder (*n*=1). See Table 1 for baseline socio-demographic and clinical characteristics.

**Table 1:** Sociodemographic and clinical characteristics of the 6 participants at baseline.

		N	%
Gender	Female	6	100.0
Age	*M (SD)*	43.3	9.5
In a relationship		6	100.0
Children		5	83.3
Educational level*	Low	1	16.7
	Middle	1	16.7
	High	4	66.7
Cancer site / type	Breast	4	66.7
	Colon	1	16,7
	Non-Hodgkin	1	16.7
Type of treatment	Chemo	6	100.0
	Operation	5	83.3
	Radiation	4	66.7
	Antihormonal	4	66,7
	Immune	2	33.4
Time since diagnosis in years	1	4	66.7
	2	1	16.7
	8	1	16.7

Note.

* Low = primary and lower secondary education; Middle = upper secondary education; High = higher vocational training and university.

### Visual analysis findings

**Figure 1:**
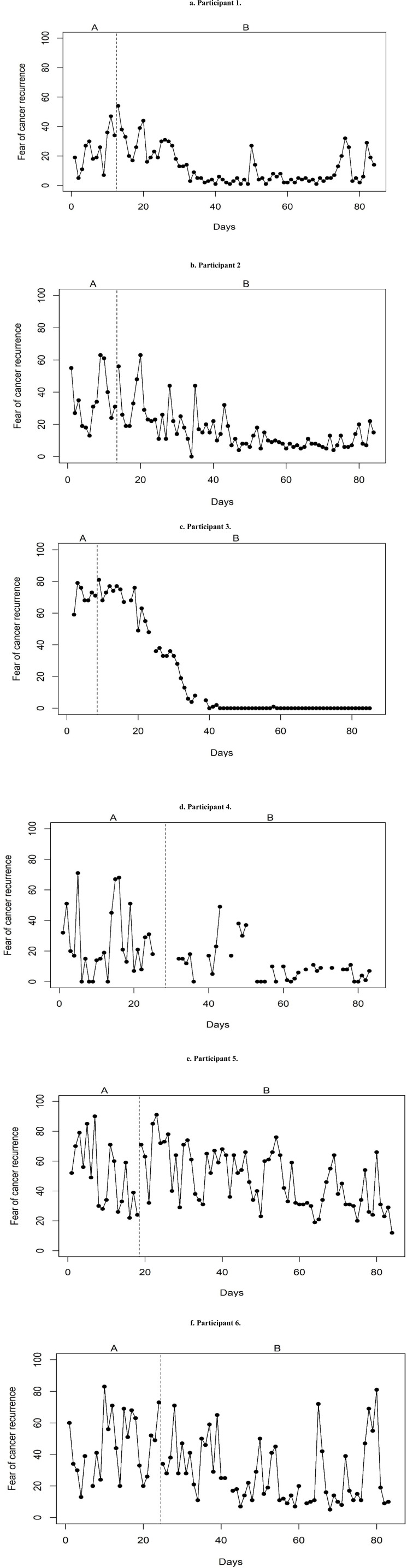
Daily level of FCR during baseline (phase A) and intervention period (phase B) for each participant.

Participant 1 reported an average FCR level of 23.25 during phase A and 12.31 during phase B (see Figure 1a and Table 2). Phase A showed high variability in scores and a modest increasing trend. Phase B showed less variability in scores: during EMDR (day 13 – 35) the variability appeared substantial while during follow-up FCR seemed to stabilize at lower levels, besides some short peaks around day 50 and 80. Despite variability, a clear decreasing trend could be noticed during EMDR treatment. After EMDR a modest increasing trend was visible: it seemed as though FCR peaked for a day, dropped and stabilized again at a low rate. A considerable overlap in scores at the end of phase A and the start of phase B suggests that the effect of treatment was not immediate but delayed.

**Table 2:** Daily FCR levels per phase for each participant.

	Phase A				Phase B			
P	Days	*M*	*(SD)*	Range	Days	EMDR days	*M*	*(SD)*	Range
1	1-12	23.25	(12.51)	5 – 47	13-84	13-34	12.31	(12.24)	1-54
2	1-13	34.69	(16.15)	13 – 63	13-84	14-48	15.72	(12.32)	0-63
3	1-8	70.57	(6.50)	59 – 79	9-84	9-36	17.08	(27.27)	0-81
4	1-28	25.32	(19.00)	0 – 71	29-84	29-53	10.78	(11.87)	0-49
5	1-18	50.39	(22.11)	22 – 90	19-84	19-60	47.68	(19.06)	12-91
6	1-30	45.17	(20.25)	13 – 83	31-84	31-51	27.84	(19.83)	5-81

*Note*. P = participant number.

The patterns of participant 2-4 (Figure 1b-1d) showed several similarities with participant 1 regarding the slight increasing trend in phase A and a two-folded trend in phase B (i.e., a decreasing trend followed by a modest increase or stabilization), the patterns of variability during the phases and the immediate overlap in scores, pointing out a delayed effect of treatment. The most obvious treatment effect was seen in participant 3: after finishing EMDR, the FCR levels reached a bottom effect, with little variance in scores.

The plots of participants 5 and 6 (Figure 1e-1f) showed different patterns. The difference in average levels between phase A and B was less obvious and the variability during both phases was large. Participant 5 showed a remarkable decreasing trend in phase A and a modest decreasing trend in phase B, with a sudden increase during the first days of EMDR, implying a worsening of fear. Participant 6 had a sudden decrease in scores during the first days of phase B, which suggests an immediate treatment effect. However, the following days and weeks showed multiple FCR peaks. In these participants, the overlap in scores between phase A and B was high and the expected treatment effect seemed less obvious.

### Regression analysis findings

For participant 1 we found significant effects of time, treatment and its interaction, indicating that the increasing trend in Phase A significantly differed from the decreasing trend in phase B (see Table 3). In participant 6, a significant effect of treatment was found, implying a significant decrease of FCR scores from phase A to B. Unexpectedly, in participant 5 we found a significant effect in the opposite direction: the treatment phase showed a less steep reduction of daily FCR levels than during the baseline phase. Among the remaining participants, no significant effects were found. HLM analysis showed no significant effects, indicating that EMDR did not result in decreased FCR levels at the group level.

**Table 3:** Regression analysis: one-level analysis for each participant and HLM (n=6)

P	Regressor	Coefficient	SE	*t*	*p*
Simple regression					
1	Intercept	37.05	6.69	5.54	<0.001
	Time	2.12	0.91	2.34	0.022
	Phase A-B	-15.16	7.15	-2.12	0.037
	Time x Phase	- 2.39	0.91	-2.63	0.010
2	Intercept	38.46	6.56	5.86	<0.001
	Time	0.54	0.83	0.65	0.517
	Phase A-B	-10.30	7.064	-1.46	0.149
	Time x Phase	- 0.89	0.829	-1.08	0.284
3	Intercept	72.86	12.67	5.75	<0.001
	Time	0.57	2.83	0.20	0.841
	Phase A-B	-16.60	13.15	-1.26	0.210
	Time x Phase	- 1.58	2.83	0.57	0.580
4	Intercept	24.25	9.26	2.62	0.011
	Time	-0.06	0.46	-0.12	0.902
	Phase A-B	-5.04	10.68	-0.47	0.639
	Time x Phase	-0.27	0.49	0.54	0.589
5	Intercept	27.85	7.99	3.48	0.001
	Time	-2.37	0.74	-3.21	0.002
	Phase A-B	38.65	8.92	4.33	<0.001
	Time x Phase	1.79	0.75	2.40	0.019
6	Intercept	53.51	8.38	6.39	<0.001
	Time	0.68	0.59	1.14	0.256
	Phase A-B	-20.10	9.84	-2.04	0.045
	Time x Phase	-0.87	0.62	-1.42	0.160
**Two-level HLM**					
*Fixed effects*		Estimate	SE	*t*	*p*
	Intercept	40.50	8.08	5.01	0.004
	Time	-0.08	0.59	-0.14	0.897
	Phase A-B	-2.70	8.67	-0.31	0.768
	Time x Phase	-0.38	0.59	-0.64	0.556
*Random effects*		SD	Corr		
	Intercept	17.74
	Time	1.03	0.493		
	Phase A-B	18.85	-0.458	-0.999	
	Time x Phase	1.06	-0.678	-0.693	0.953
*Residual*	15.16			

*Note*. P = participant number.

### Randomization test findings

For none of the participants, the RT showed a significant effect between phase A and B, indicating that the introduction of EMDR did not lead to an immediate decrease in FCR (see Table 4). The combined *p*-value of 0.845 confirmed these null-findings.

**Table 4:** Randomization test for each participant and combined (n=6).

Participant	MA-MB	*p*-value
1	10.94	0.833
2	18.97	0.700
3	53.50	0.700
4	14.54	0.300
5	-2.71	1.000
6	17.33	0.200
Combined		0.845

*Note.* MA = mean level of FCR in phase A;

MB = mean level of FCR in phase B

### Reliable change

Participants 3-6 showed a clinically relevant decrease according to the RCI (Table 5). Participants 1 and 2 showed a decrease of 5, just below the reliable change cut-off of 6. All participants scored below the clinical cut-off of 22 at T3. The FCR decrease was therefore considered clinically relevant for participants 3-6.

**Table 5:** FCRI-SF scores at each time point.

P	FCRI-SF				
	T0	T1	T2	T3	T1-T3
1	25	23	missing	18	5
2	26	25	17	20	5
3	28	29	11	12	17*
4	23	22	10	10	13*
5	25	23	16	15	8*
6	23	22	10	14	9*

*Note.* FCRI-SF T0: start phase A, T1: start phase B; T2: end EMDR; T3: end phase B.

* Clinically relevant change: RCI of ≥6 and FCRI score <22.

### Qualitative findings

In the semi-structured interviews, participants only reported positive changes since EMDR treatment. No worsening of FCR or negative side-effects were mentioned, despite the explicit question about negative experiences with EMDR.

### Being less overwhelmed by fear

Participants described how FCR did not vanish but was less overwhelming: “It does not make me nauseous anymore” or “these thoughts are no longer on my mind all day”. Participants mentioned they felt more relaxed and had “more peace of mind”. One person stated: “It’s as if my fears are now stored in boxes with lids, which I can control in opening”. As participants were better able to manage their fear, they experienced more space to reflect on other important areas of their life, such as work and family.

### Putting fear in perspective

Several participants described how they can more easily put things in perspective when experiencing FCR. Their future holds more options than their worst-case scenario: “It [fear] is based on nothing. I have been ill of course, but chances are it will keep going well, and that has been proved by now, because it has been two years. Everything is okay.” Some participants explained how they were able to keep their distance from other people’s suffering rather than relating it to themselves: “I am able to think now, this is a very sad situation for the other person, but it’s not about me”.

### Feeling empowered by facing one’s fear

Most participants experienced the FF as a very intense part of treatment, but also as empowering because it helped them to face their worst fears: “That was the most intense part. Normally I distract myself, start walking, or drawing. But now I faced it. It’s not completely gone, but I can handle it”. As part of the procedure, participants described their worst fears to their therapist. As a result, they were better able to verbalize their fears and felt more competent in sharing their fear with others.

### Therapist experiences

Therapists valued the combination of desensitization of memories with the FF. They explained that participants seemed relieved once they had desensitized painful memories or frightening future images related to their illness. One therapist mentioned: “My participant realized, this is not happening now, but in the future. I did everything I could, it’s okay and my partner will manage without me”. The treatment was experienced as efficient and helped therapists to focus on FCR.

## Discussion

The present study explored whether EMDR with FF is helpful in treating FCR among cancer survivors. After completing EMDR, all participants described positive changes in mental wellbeing and daily functioning during the interview. They were better able to cope with FCR. Visual analysis of daily FCR levels prior, during and after EMDR treatment is in line with this positive evaluation. Quantitative analyses resulted in mixed findings. Based on regression analysis, two out of six participants appeared to improve; unexpectedly one participant deteriorated. Based on the randomization test, none of the participants appeared to improve. Based on the reliable change index, four participants appeared to improve. The discrepancy among quantitative findings can be related to multiple sources. Firstly, a difference in outcome measures could explain the discrepancy. For the daily assessment of FCR we asked participants how much FCR they had experienced in the past 24 hours. However, from the qualitative interviews, we discovered that high levels of FCR do not directly reflect serious impairment through FCR. Patients described they still experienced high levels of FCR from time to time after EMDR, but were less affected by it because the FCR was less unexpected, less long-lasting and better to handle because their coping had improved. By contrast, the FCRI-SF, which was used to assess reliable change, does not only measure the level of FCR, but also how often and how long they worry and whether it triggers unpleasant thoughts. In line with the interview findings, all participants scored beneath the clinical range of 22 on the FCRI-SF at follow-up. In order to capture how burdened patients are by FCR, the daily assessments should not only assess the severity but also the frequency and duration of FCR. Items of the newly developed Ottawa Clinical Fear of Recurrence – Self-report (OCFR-SF) seem ideally suited for such daily assessments as it contains items on severity, functional impairment and intrusions (Giguère et al., 2024).

Secondly, we unexpectedly encountered trends and high variability in daily FCR levels, limiting the power of the regression analysis and RT. This was surprising as multiple studies have reported that clinical FCR does not change over time without intervention (Simard et al., 2013). This suggests that a higher frequency of measurements (i.e., daily instead of monthly) can paint a more nuanced picture of the course of FCR.

Thirdly, the RT assumes an immediate effect between phase A and B, making it a less suitable analysis approach to study the effect of EMDR. EMDR has the potential to cause increased anxiety or fatigue during the three days following a session (De Jongh et al., 2018). The analysis could not account for this delayed effect. Comparing the baseline phase to the follow-up phase rather than the treatment phase might have been more suitable to detect potential differences, although it is most likely that the largest symptom decrease occurs somewhere during treatment. In light of these alternative explanations, and the positive evaluation of the visual analysis and patients’ experiences, we consider EMDR with FF as a promising treatment for cancer patients suffering from FCR. These findings are in line with results from Bruin et al. (2023), who found a significant decrease in FCR after EMDR with FF in cancer patients.

Zooming in on participants’ experiences, EMDR seems especially promising in managing two key characteristics of FCR: intrusions and death anxiety (Berlin & von Blanckenburg, 2022; Curran et al., 2020; Smith et al., 2018). Reduction of intrusions is a common finding in EMDR studies in PTSD (Carletto et al., 2019; Khan et al., 2018). By adding FF to EMDR, death anxiety became an important part of treatment. Earlier research suggests that inadequate coping with death anxiety can maintain FCR (Berlin & von Blanckenburg, 2022; Sharpe et al., 2018). According to Terror Management Theory (TMT) humans strive to cope with this intense fear mainly by two defense mechanisms: a more proximal defense mechanism that is marked by behaviors such as suppressing thoughts, denial or health behavior, and a more distal defense mechanism oriented at finding meaning and value in life (Pyszczynski et al., 1999). This distal defense includes behaviors that promote self-esteem, often targeted at identifying and living according to one’s values (Sharpe et al., 2018). Long-term avoidance of death anxiety by suppression or denial often corresponds with intrusive thoughts, or other fears (Menzies et al., 2019). Facing these fears is considered helpful in increasing more distal ways of coping, by (re)finding and creating meaning in life (Yalom, 2008). Using FF, fearful images of death and dying are desensitized and assigned with less threatening meaning (De Jongh & Ten Broeke, 2019). Several participants described a process that corresponds with a change from a proximal to a distal defense mechanism through EMDR: the confrontation with their worst fears was difficult, but gradually they experienced more space that enabled them to make choices towards a more meaningful life.

Previous studies have shown that reduction of intrusions after EMDR among patients with PTSD has been correlated with improvements in cognitive and emotional functioning, including normalization of brain functioning in limbic and prefrontal areas (Capezzani et al., 2013; Khan et al., 2018; Shapiro, 1989). These findings imply that improvement of managing death anxiety by EMDR, cannot only reduce emotional suffering but also increase responsivity to further treatment. Such interventions could target other aspects of FCR, such as excessive worrying and threat appraisals (e.g., Cognitive Behavioral Therapy, Metacognitive Therapy) and help participants live a meaningful life (e.g., Acceptance and Commitment Therapy).

### Methodological Issues

While this SCED study suggests that EMDR is a promising treatment for clinical FCR, the following methodological issues warrant consideration. Firstly, while we followed recommended numbers of baseline observations, randomi-zation points and follow-up assessments for SCEDs, these numbers were rather small and appeared insufficient to account for trends and variability in the data, limiting the power of our analyses (Moeyaert et al., 2014). More complex regression models could have fitted the data better, but were not applied with respect to the modest number of observations in some of the follow-up phases (Manolov & Moeyaert, 2017; Michiels & Onghena, 2018). As the RT assumes an immediate effect, comparing baseline to end rather than start of treatment might have been better suited to assess the effect of EMDR. Furthermore, the analysis of qualitative data seemed enriching for clinical and theoretical purposes, but note that data saturation was not reached and coding was done by one researcher. Secondly, due to the lack of an active control group we cannot exclude the possibility that achieved benefits are partly the consequence of nonspecific (‘placebo’) factors common to all psychotherapies (Perkins & Rouanzoin, 2002). However, among patients with PTSD, the literature has shown that treatment effects of EMDR are much larger and longer lasting than placebo effects (De Jongh et al., 2024). Thirdly, the follow-up period varied between 2 to 7 weeks among participants. Future randomized controlled trials should include at least a 1-year follow-up period to study the long-term effects of EMDR. Lastly, as in any SCED, generalizability of findings is limited. While our sample showed some diversity in types of cancer, treatment and years since diagnosis, only women were included.

### Conclusion

In sum, mixed results were found in our SCED on EMDR with FF on clinical FCR. As clinical FCR is considered a multidimensional construct (Curran et al., 2020; Mutsaers et al., 2020), various types of psychological treatment using different foci have been modestly effective in managing FCR (Tauber et al., 2019). What combination of treatment elements would fit the individual remains unclear. Further research is needed to establish the effectiveness of EMDR on FCR. By also assessing perpetuating factors, such as intrusions, death anxiety and cognitive functioning, we gain more insight in the working mechanisms of EMDR on FCR. If research confirms that EMDR with the FF is effective in changing proximal to distal coping with death anxiety, it could be a valuable start of treatment for patients who suffer from intrusions and death anxiety to use a more personalized approach to FCR.

## Data Availability

The data that support the findings of this study are available on request from the corresponding author MS.

## Action editor

Lars-Gunnar Lundh served as action editor for this article.

## Open access

This article is distributed under the terms of the Creative Commons Attribution 4.0 International License (http://creativecommons.org/licenses/by/4.0/), which permits unrestricted use, distribution, and reproduction in any medium, provided you give appropriate credit to the original author(s) and the source, provide a link to the Creative Commons license, and indicate if changes were made.

## References

[cit0001] Almeida, S. N., Elliott, R., Silva, E. R., & Sales, C. M. D. (2019). Fear of cancer recurrence: A qualitative systematic review and meta-synthesis of patients’ experiences. *Clinical Psychology Review*, 68, 13–24. 10.1016/j.cpr.2018.12.001 30617013

[cit0002] Berlin, P., & von Blanckenburg, P. (2022). Death anxiety as general factor to fear of cancer recurrence. *Psycho-Oncology*, 31(9), 1527–1535. 10.1002/pon.5974 35665981

[cit0003] Bisseling, E. M., Compen, F. R., Schellekens, M. P. J., Thewes, B., Speckens, A. E. M., & van der Lee, M. L. (2021). Exploring fear of cancer recurrence in a sample of heterogeneous distressed cancer patients with and without a psychiatric disorder. *Journal of Clinical Psychology in Medical Settings*, 28(3), 419 – 426. 10.1007/s10880-021-09776-2 34138447 PMC8458175

[cit0004] Braun, V., & Clarke, V. (2006). Using thematic analysis in psychology. *Qualitative Research in Psychology*, 3, 77–101. 10.1191/1478088706qp063oa

[cit0005] Bruin, J., van Rood, Y. R., Peeters, K. C. M. J., de Roos, C., Tanious, R., Portielje, J. E. A., Gelderblom, H., & Hinnen, S. C. H. (2023). Efficacy of eye movement desensitization and reprocessing therapy for fear of cancer recurrence among cancer survivors: a randomized single-case experimental design. *European Journal of Psychotraumatology*, 14(2). 10.1080/20008066.2023.2203427 PMC10165926 37144665

[cit0006] Bulté, I., & Onghena, P. (2009). Randomization tests for multiple-baseline designs: An extension of the SCRT-R package. *Behavior Research Methods*, 41(2), 477–485. 10.3758/BRM.41.2.477 19363188

[cit0007] Bulté, I., & Onghena, P. (2013). The single-case data analysis package: Analysing single-case experiments with R software. *Journal of Modern Applied Statistical Methods*, 12(2). 10.22237/jmasm/138328002

[cit0008] Capezzani, L., Ostacoli, L., Cavallo, M., Carletto, S., Fernandez, I., Solomon, R., Pagani, M., & Cantelmi, T. (2013). EMDR and CBT for cancer patients: Comparative study of effects on PTSD, anxiety, and depression. *Journal of EMDR Practice and Research*, 7(3), 134–143. 10.1891/1933-3196.7.3.134

[cit0009] Carletto, S., Porcaro, C., Settanta, C., Vizzari, V., Stanizzo, M. R., Oliva, F., Torta, R., Fernandez, I., Moja, M. C., Pagani, M., & Ostacoli, L. (2019). Neurobiological features and response to eye movement desensitization and reprocessing treatment of posttraumatic stress disorder in patients with breast cancer. European *Journal of Psychotraumatology*, 10(1). 10.1080/20008198.2019.1600832 PMC6495116 31073391

[cit0010] Curran, L., Sharpe, L., & Butow, P. (2017). Anxiety in the context of cancer: A systematic review and development of an integrated model. *Clinical Psychology Review*, 56, 40–54. 10.1016/j.cpr.2017.06.003 28686905

[cit0011] Curran, L., Sharpe, L., MacCann, C., & Butow, P. (2020). Testing a model of fear of cancer recurrence or progression: the central role of intrusions, death anxiety and threat appraisal. *Journal of Behavioral Medicine*, 43(2), 225–236. 10.1007/s10865-019-00129-x 31907743

[cit0012] de Jongh, A., de Roos, C., & El-Leithy, S. (2024). State of the science: Eye movement desensitization and reprocessing (EMDR) therapy. *Journal of Traumatic Stress*, 37(2), 205–216. 10.1002/jts.23012 38282286

[cit0013] de Jongh, A., Ernst, R., Marques, L., & Hornsveld, H. (2013). The impact of eye movements and tones on disturbing memories involving PTSD and other mental disorders. *Journal of Behavior Therapy and Experimental Psychiatry*, 44(4). 10.1016/j.jbtep.2013.07.002 23892070

[cit0014] de Jongh, A & Ten Broeke, E. (2019). *Handboek EMDR: Een geprotocolleerde behandelmethode voor de gevolgen van psychotrauma*. (7th ed.). Pearson Benelux. https://www.pearsonclinical.nl/handboek-emdr

[cit0015] Declercq, L., Cools, W., Beretvas, S. N., Moeyaert, M., Ferron, J. M., & van den Noortgate, W. (2020). MultiSCED: A tool for (meta-)analyzing single-case experimental data with multilevel modeling. *Behavior Research Methods*, 52(1), 177–192. 10.3758/s13428-019-01216-2 30972557

[cit0016] Engelhard, I. M., van den Hout, M. A., Dek, E. C. P., Giele, C. L., van der Wielen, J. W., Reijnen, M. J., & van Roij, B. (2011). Reducing vividness and emotional intensity of recurrent “flashforwards” by taxing working memory: An analogue study. *Journal of Anxiety Disorders*, 25(4), 599–603. 10.1016/j.janxdis.2011.01.009 21376527

[cit0018] Engelhard, I. M., van den Hout, M. A., Janssen, W. C., & van der Beek, J. (2010). Eye movements reduce vividness and emotionality of “ flashforwards.” *Behaviour Research and Therapy*, 48(5), 442–447. 10.1016/j.brat.2010.01.003 20129601

[cit0019] Fardell, J. E., Thewes, B., Turner, J., Gilchrist, J., Sharpe, L., Smith, A. Ben, Girgis, A., & Butow, P. (2016). Fear of cancer recurrence: a theoretical review and novel cognitive processing formulation. *Journal of Cancer Survivorship*, 10(4), 663–673. 10.1007/s11764-015-0512-5 26782171

[cit0020] Giguère, L., Mutsaers, B., Harris, C., Smith, A. ‘Ben,’ Humphris, G. M., Costa, D., Kogan, C. S., Simard, S., & Lebel, S. (2024). The Ottawa clinical fear of recurrence instruments: A screener, self-report, and clinical interview. *Psycho-Oncology*, 33(6). 10.1002/pon.6364 38824493

[cit0021] Heyvaert, M., Moeyaert, M., Verkempynck, P., van den Noortgate, W., Vervloet, M., Ugille, M., & Onghena, P. (2017). Testing the Intervention Effect in Single-Case Experiments: A Monte Carlo Simulation Study. *Journal of Experimental Education*, 85(2), 175–196. 10.1080/00220973.2015.1123667

[cit0022] Heyvaert, M., & Onghena, P. (2014). Randomization tests for single-case experiments: State of the art, state of the science, and state of the application. *Journal of Contextual Behavioral Science*, 3(1), 51–64. 10.1016/j.jcbs.2013.10.002

[cit0023] Hornsveld, H., de Jongh, A., & ten Broeke, E. (2018). Verschillen tussen het Nederlandse EMDR-standaardprotocol en het originele protocol van Shapiro. Deel IV: Scherpstellen, desensitisatie en ‘Back to target’. *EMDR Magazine*, 6(16), 33–36.

[cit0024] Horst, F., den Oudsten, B., Zijlstra, W., de Jongh, A., Lobbestael, J., & de Vries, J. (2017). Cognitive behavioral therapy vs. eye movement desensitization and reprocessing for treating panic disorder: A randomized controlled trial. *Frontiers in Psychology*, 8. 10.3389/fpsyg.2017.01409 PMC5563354 28868042

[cit0025] Huitema, B. E., & McKean, J. W. (1998). Irrelevant Autocorrelation in Least-Squares Intervention Models. *Psychological Methods*, 3(1). 10.1037/1082-989X.3.1.104

[cit0026] Jacobson, N. S., & Truax, P. (1991). Clinical Significance: A Statistical Approach to Defining Meaningful Change in Psychotherapy Research. *Journal of Consulting and Clinical Psychology*, 59(1). 10.1037/0022-006X.59.1.12 2002127

[cit0027] Jarero, I., Givaudan, M., & Osorio, A. (2018). Randomized controlled trial on the provision of the EMDR Integrative Group Treatment Protocol adapted for ongoing traumatic stress to female patients with cancer-related posttraumatic stress disorder symptoms. *Journal of EMDR Practice and Research*, 12(3), 94–104. 10.1891/1933-3196.12.3.94

[cit0028] Khan, A. M., Dar, S., Ahmed, R., Bachu, R., Adnan, M., & Kotapati, V. P. (2018). Cognitive Behavioral Therapy versus Eye Movement Desensitization and Reprocessing in Patients with Post-traumatic Stress Disorder: Systematic Review and Metaanalysis of Randomized Clinical Trials. *Cureus*, 10(9): e3250 10.7759/cureus.3250 30416901 PMC6217870

[cit0029] Kratochwill, T. R., Hitchcock, J., Horner, R. H., Levin, J. R., Odom, S. L., Rindskopf, D. M., & Shadish, W. R. (2010). Single-case designs technical documentation. Retrieved from What Works Clearinghouse website: http://ies.ed.gov/ncee/wwc/pdf/wwc_scd.pdf.

[cit0030] Kratochwill, T. R., & Levin, J. R. (2010). Enhancing the Scientific Credibility of Single-Case Intervention Research: Randomization to the Rescue. *Psychological Methods*, 15(2), 124–144. 10.1037/a0017736 20515235

[cit0032] Lebel, S., Ozakinci, G., Humphris, G., Mutsaers, B., Thewes, B., Prins, J., Dinkel, A., & Butow, P. (2016). From normal response to clinical problem: definition and clinical features of fear of cancer recurrence. *Supportive Care in Cancer*, 24(8). 10.1007/s00520-016-3272-5 27169703

[cit0033] Logie, R., & de Jongh, A. (2018). *The Flashforward Procedure. In Eye Movement Desensitization and Reprocessing EMDR Therapy Scripted Protocols and Summary Sheets*. Springer Publishing Company. 10.1891/9780826131683.0003

[cit0034] Luigjes-Huizer, Y. L., Tauber, N. M., Humphris, G., Kasparian, N. A., Lam, W. W. T., Lebel, S., Simard, S., Smith, A. ben, Zacha-riae, R., Afiyanti, Y., Bell, K. J. L., Custers, J. A. E., de Wit, N. J., Fisher, P. L., Galica, J., Garland, S. N., Helsper, C. W., Jeppesen, M. M., Liu, J., … van der Lee, M. L. (2022). What is the prevalence of fear of cancer recurrence in cancer survivors and patients? A systematic review and individual participant data meta-analysis. *Psycho-Oncology*, 31(6), 879–892. 10.1002/pon.5921 35388525 PMC9321869

[cit0035] Manly, B. F. J. (2007). Randomization Tests, 4th Edition by Eugene S. Edgington, Patrick Onghena. *International Statistical Review*, 75(2). 10.1111/j.1751-5823.2007.00015_21.x

[cit0036] Manolov, R., & Moeyaert, M. (2017). Recommendations for Choosing Single-Case Data Analytical Techniques. *Behavior Therapy*, 48(1), 97–114. 10.1016/j.beth.2016.04.008 28077224

[cit0037] Menzies, R. E., Sharpe, L., & Dar-Nimrod, I. (2019). The Relationship between death anxiety and severity of mental illnesses. *British Journal of Clinical Psychology*, 58(4), 452–467. 10.1111/bjc.12229 31318066

[cit0038] Michiels, B., & Onghena, P. (2018). Randomized single-case AB phase designs: Prospects and pitfalls. *Behavior Research Methods*, 51(6), 2454–2476. 10.3758/s13428-018-1084-x 30022457

[cit0039] Moeyaert, M., Ferron, J. M., Beretvas, S. N., & van den Noortgate, W. (2014). From a single-level analysis to a multi-level analysis of single-case experimental designs. *Journal of School Psychology*, 52(2), 191–211. 10.1016/J.JSP.2013.11.003 24606975

[cit0040] Mutsaers, B., Butow, P., Dinkel, A., Humphris, G., Maheu, C., Ozakinci, G., Prins, J., Sharpe, L., Smith, A. “Ben,” Thewes, B., & Lebel, S. (2020). Identifying the key characteristics of clinical fear of cancer recurrence: An international Delphi study. *Psycho-Oncology*, 29(2), 430–436. 10.1002/pon.5283 31713279

[cit0041] Onghena, P., Maes, B., & Heyvaert, M. (2019). Mixed Methods Single Case Research: State of the Art and Future Directions. *Journal of Mixed Methods Research*, 13(4), 461–480. 10.1177/1558689818789530

[cit0042] Perkins, B.R. & Rouanzoin, C.C. (2001). A critical evaluation of current views regarding eye movement desensitization and reprocessing (EMDR): Clarifying points of confusion. *Journal of Clinical Psychology*, 58(1), 77–97. 10.1002/jclp.1130 11748598

[cit0043] Pyszczynski, T., Greenberg, J., & Solomon, S. (1999). A dual-process model of defense against conscious and unconscious death-related thoughts: An extension of terror management theory. *Psychological Review*, 106(4), 835–845. 10.1037/0033-295X.106.4.835 10560330

[cit0044] Shapiro, F. (2001). *Eye Movement Desensitization and Reprocessing Basic Principles, Protocols and Procedures* (2nd edition). Guilford Press. https://www.guilford.com/books/Eye-Movement-Desensitization-and-Reprocessing-EMDR-Therapy/Francine-Shapiro/9781462532766?srsltid=AfmBOorn-sYMfnWv8H7Dac4K3s6FXOm5E7jgmZen323WiOziR-WlCZMkuq

[cit0045] Shapiro, F, 1989. (1989). Efficacy of the eye movement desensitization procedure in the treatment of traumatic memories. *Journal of Traumatic Stress*, 2, 199–223. 10.1002/jts.2490020207

[cit0046] Sharpe, L., Curran, L., Butow, P., & Thewes, B. (2018). Fear of cancer recurrence and death anxiety. *Psycho-Oncology*, 27(11), 2559–2565. 10.1002/pon.4783 29843188

[cit0047] Simard, S., & Savard, J. (2009). Fear of Cancer Recurrence Inventory: Development and initial validation of a multidimensional measure of fear of cancer recurrence. *Supportive Care in Cancer*, 17(3), 241–251. 10.1007/s00520-008-0444-y 18414902

[cit0048] Simard, S., Thewes, B., Humphris, G., Dixon, M., Hayden, C., Mireskandari, S., & Ozakinci, G. (2013). Fear of cancer recurrence in adult cancer survivors: a systematic review of quantitative studies. *Journal of Cancer Survivorship*, 7(3), 300–322. 10.1007/s11764-013-0272-z 23475398

[cit0049] Smith, A. ‘Ben,’ Sharpe, L., Thewes, B., Turner, J., Gilchrist, J., Fardell, J. E., Girgis, A., Tesson, S., Descallar, J., Bell, M. L., Beith, J., Butow, P., Beatty, L., Bennett, B., Brebach, R., Brock, C., Butler, S., Byrne, D., Day, S., … Watt, P. (2018). Medical, demographic and psychological correlates of fear of cancer recurrence (FCR) morbidity in breast, colorectal and melanoma cancer survivors with probable clinically significant FCR seeking psychological treatment through the ConquerFear study. *Supportive Care in Cancer*, 26(12), 4207–4216. 10.1007/s00520-018-4294-y 29882025

[cit0050] Tarquinio, C., Machado, J., Bruno, J., & Gendarme, S. (2022). The Treatment of Anxious-Depressive Disorders among Breast Cancer Patients Integrating the EMDR psychotherapy. *Psychology*, 13, 313–327. 10.4236/psych.2022.133019

[cit0051] Tauber, N. M., O’toole, M. S., Dinkel, A., Galica, J., Humphris, G., Lebel, S., Maheu, C., Ozakinci, G., Prins, J., Sharpe, L., Ben, A. “, Smith, ”, Thewes, B., Simard, S., & Zachariae, R. (2019). Effect of Psychological Intervention on Fear of Cancer Recurrence: A Systematic Review and Meta-Analysis. *Journal of Clinical Oncology*, 37(31), 2899–2915. 10.1200/JCO.19.00572 31532725 PMC6823887

[cit0052] van den Noortgate, W., & Onghena, P. (2003). Hierarchical linear models for the quantitative integration of effect sizes in single-case research. *Behavior Research Methods, Instruments, and Computers*, 35(1), 1–10. 10.3758/BF03195492 12723775

[cit0053] van Helmondt, S. J., van der Lee, M. L., Bisseling, E. M., Lodder, P., & de Vries, J. (2021). Factor structure of the Fear of Cancer Recurrence Inventory (FCRI): Comparison of international FCRI factor structure data and factor analysis of the Dutch FCRI-NL using three predominantly breast cancer samples. *European Journal of Cancer Care*, 30(5). 10.1111/ecc.13431 PMC8519082 33763943

[cit0054] van Helmondt, S. J., van der Lee, M. L., & de Vries, J. (2017). Translation and validation of the Dutch version of the Fear of Cancer Recurrence Inventory (FCRI-NL). *Journal of Psychosomatic Research*, 102, 21–28. 10.1016/j.jpsychores.2017.09.001 28992893

[cit0055] Yalom, I. D. (2008). Staring at the sun: Overcoming the terror of death. *Humanistic Psychologist*, 36, 3–4. 10.1080/08873260802350006

[cit0056] Yunitri, N., Kao, C. C., Chu, H., Voss, J., Chiu, H. L., Liu, D., Shen, S. T. H., Chang, P. C., Kang, X. L., & Chou, K. R. (2020). The effectiveness of eye movement desensitization and reprocessing toward anxiety disorder: A meta-analysis of randomized controlled trials. *Journal of Psychiatric Research*, 123, 102–113. 10.1016/j.jpsychires.2020.01.005 32058073

